# Cosolvent Control of Lower and Upper Critical Solution Behavior in Polyelectrolyte Complexes

**DOI:** 10.1021/acsmacrolett.5c00315

**Published:** 2025-07-01

**Authors:** Yuanchi Ma, Vivek M. Prabhu

**Affiliations:** † College of Polymer Science and Engineering, 66280 Qingdao University of Science and Technology, Qingdao, Shandong 266042, China; ‡ Materials Science and Engineering Division, Material Measurement Laboratory, 10833 National Institute of Standards and Technology, 100 Bureau Drive, Gaithersburg, Maryland 20899-8542, United States

## Abstract

We report that polar cosolvent–water mixtures offer a unique approach to controlling the liquid–liquid phase separation (LLPS) of polyelectrolyte complex solutions formed from degree of polymerization-matched mixtures of strong and weak polyelectrolytes—respectively, quaternary poly­(*N,N*-dimethylaminoethyl methacrylate chloride) (qPDMAEMA) and sodium poly­(acrylate) (PA). As observed in prior work, associative LLPS in water exhibits an upper-critical salt concentration with stoichiometric complexes and lower-critical solution temperature (LCST) behavior, where electrostatic correlations are believed to drive phase behavior. However, upon addition of a miscible cosolvent prior to mixing the individual polyelectrolytes at room temperature, we observe a shift in the LCST and the appearance of an upper-critical solution temperature (UCST). This new UCST feature corresponds to a segregative LLPS, whereby the polycation partitions out of the polyanion-rich dense phase and into the supernatant. This behavior arises with cosolvents that decrease (e.g., ethylene glycol) or increase (e.g., *N*-methyl formamide) the average solvent dielectric constant, suggesting that electrostatic correlations may not primarily control the phase behavior for cosolvated coacervate systems. A conceptual 3D phase surface summarizing these observations for the cosolvated system suggests that two distinct surfaces with critical lines appear on the polymer–salt–temperature phase diagram.

## Graphical abstract

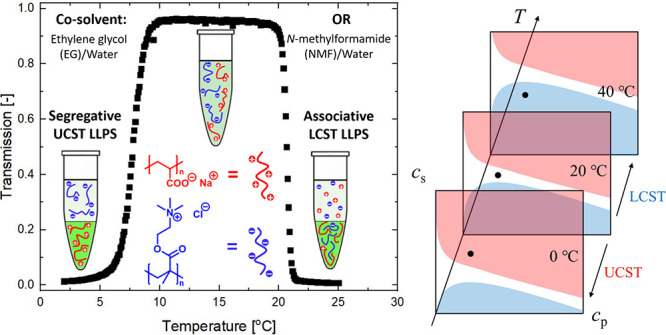

Polyelectrolyte complex (PEC) coacervation is of interest in both biophysics, where liquid–liquid phase separation (LLPS) leads to membraneless organelles, and advanced material applications, with efforts predominantly covering the effects of added salt,^1–11^ charge density of the polyelectrolytes,^9–11^ and occasionally temperature.^12–15^ The propensity of a PEC to resist dissolution is often referred to as “salt resistance”—PEC dissolution is favored at high salt concentration, low polyelectrolyte charge density, and low temperature as a result of increased charge-screening by salt ions or decreased electrostatic attraction between polyions.^16,17^ Solvent quality proves to be another way of tuning PEC solubility, although quantifying this effect in terms of interaction parameters remains a challenge.^18,19^ Nonaqueous solvents, such as deep eutectics^20^ and fluorinated alcohols,^21^ can increase the solubility and processability of PEC under low salt conditions. Salt (salinity)^22–24^ and pH shifts^25–27^ in aqueous PEC solutions are used to form porous and biocatalytic membranes in a manner similar to non-solvent-induced phase separation. PEC in nonaqueous or aqueous miscible cosolvent mixtures may offer additional pathways to form membranes. In hydrogen-bonding systems, the effects of cosolvents lead to numerous unexpected results, termed “co-nonsolvency”, whereby mixtures of miscible good solvents induce a coil–globule transition, gel–collapse transition, and decreased miscibility.^28,29^ With charged polymers, this effect is complicated by added salt and electrostatic interactions.

We narrow this knowledge gap by systematically studying the phase behavior of sodium poly­(acrylate)/quaternary poly­(*N,N*-dimethylaminoethyl methacrylate chloride) (NaPA/qPDMAEMACl) stoichiometric complexes with polar protic cosolvents of ethylene glycol (EG)/water and *N*-methylformamide (NMF)/water. The polyelectrolytes (Table S1) are nearly degree of polymerization matched with relative number-average molar mass (*M*_n_) for NaPA (*M*_n_ = 20.2 kg/mol) and qPDMAEMACl (*M*_n_ = 46.3 kg/mol). In comparison to prior work, the current system features the interplay of associative LLPS and salting-out of one polyelectrolyte, NaPA, that substantially opens opportunities in separation science via a segregative LLPS mechanism via temperature (*T*) and salt concentration (*c*_s_). The latter was reported as a salt-dependent phase re-entry.^30^

Poly­(acrylic acid) (PAA) is a weak polyelectrolyte since the acid dissociation/charge density depends on the pH of the solution. The PAA was neutralized by a strong base (NaOH) to provide an initial 100% by mol charged polymer to allow for direct comparison among various solvent compositions, which is different from using a pH-buffered solution.^1,2,9–11,31^ qPDMAEMACl is a strong polyelectrolyte.

Polyelectrolyte complex solutions of NaPA/qPDMAEMACl in EG/water, with *c*_p_ = [PA^–^] + [qPDMAEMA^+^] fixed at 0.10 mol/L, were studied for their cloud point temperature behavior. The *c*_s_ was adjusted to make the phase transition within a reasonable *T* range, which occurs near 0.50 mol/L for all of the solvent compositions studied. As shown in Figure 1a, the PECs in pure water and the 90/10 (by volume) water/EG mixture (denoted as ϕ_EG_ = 0.10) both show lower-critical solution temperature (LCST) transitions through a sharp decrease in laser transmission around 20 °C from an initial homogeneous solution. This is similar to measurements on the strong polyanion–strong polycation system of potassium poly­(styrenesulfonate)/poly­(diallyldimethylammonium bromide) (KPSS/PDADMAB) complex, where the LCST behavior corresponds to associative LLPS.^12,13^ In that case, the decrease in the dielectric constant (ε) of water upon heating leads to an increasing Bjerrum length, rather than a decrease, thereby enhancing the electrostatic correlation that leads to the LCST. The Bjerrum length (*l*_B_) is a key parameter when considering charge and dipole–dipole correlations, where *l*_B_ = *e* ^2^/(4πεε_o_ *k*_B_ *T*) and *e* is the elementary charge, *k*_B_ is Boltzmann’s constant, and ε_o_ is the permittivity of free space. Including this fact can explain many experimental features when using models that go beyond the standard Voorn–Overbeek theory (VOT).^32–34^

**Figure 1:**
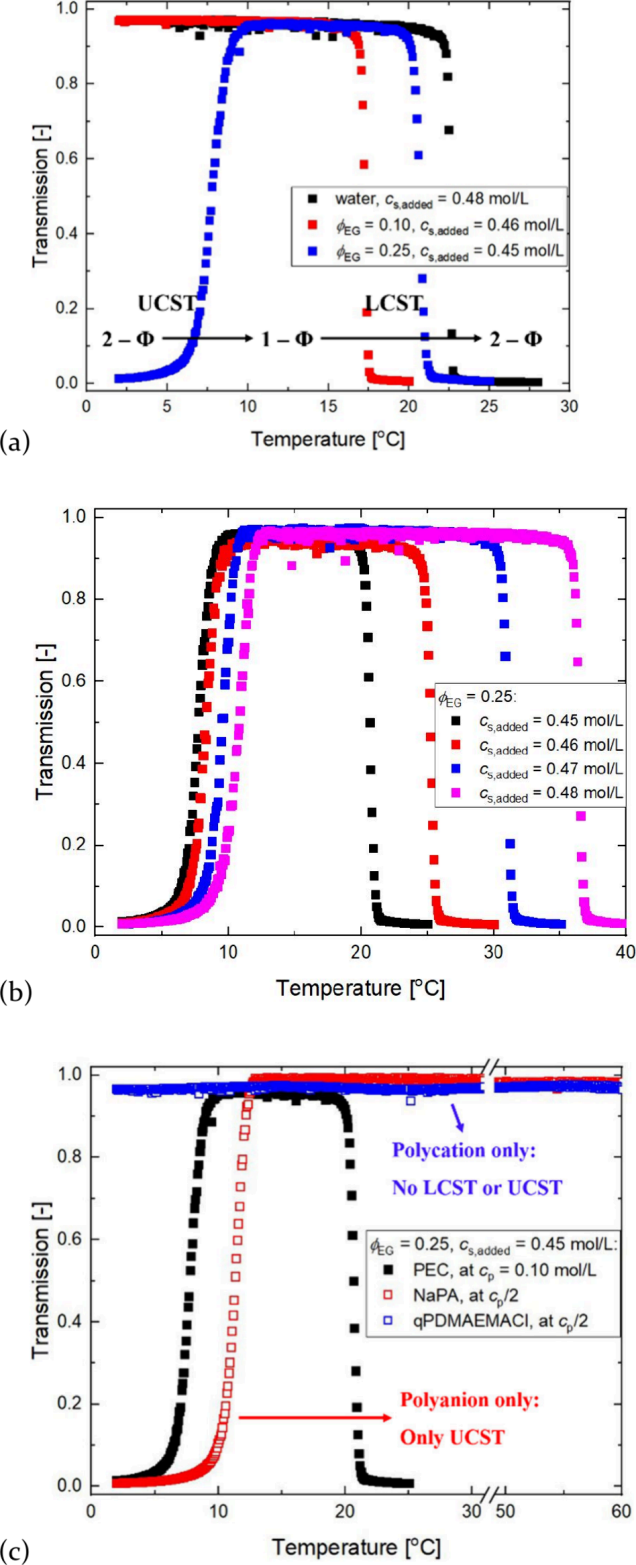
Transmission of (a) NaPA/qPDMAEMACl complexes in EG/water mixtures with ϕ_EG_ ranging from 0 to 0.25; (b) NaPA/qPDMAEMACl complexes in a fixed ϕ_EG_ for varying *c*_s_; (c) NaPA/qPDMAEMACl complex juxtaposed with NaPA and qPDMAEMACl in a single composition of solvent and salt. In all cases, [PA^–^] = [qPDMAEMA^+^] = 0.05 mol/L.

However, at ϕ_EG_ = 0.25, an additional transition appears at 7 °C that corresponds to a upper-critical solution temperature (UCST) behavior. Both UCST and LCST depend on *c*_s_ at fixed solvent composition (Figure 1b), but LCST is more sensitive to the change in *c*_s_ than UCST, showing more than a 15 °C shift with 3 mmol/L change in added *c*_s_. Similar observations were made with polar protic NMF as the cosolvent for ϕ_NMF_ = 0.10 (Figure S4c). As cosolvent content continues to increase in both cases (ϕ_EG_ > 0.25 and ϕ_NMF_ > 0.10), the one-phase (1-Φ) region vanishes and the polyelectrolytes become phase-separated at all *c*_s_. This implies that the UCST merges with the LCST miscibility gap due to cosolvent addition.

The origin of the segregative nature of the UCST is partially revealed by examining the solubility of the individual polyanion and polycation at identical ϕ_EG_ = 0.25 and *c*_s_ = 0.45 mol/L where dual criticality is observed (Figure 1c). Over the entire *T* range of interest, qPDMAEMACl shows no sign of phase separation, implying good solvent behavior throughout. However, NaPA shows a UCST at a slightly higher temperature than the corresponding polyelectrolyte complex solution. Fully ionized PAA in pure water does not phase-separate in the presence of monovalent salts, even at saturation;^35–37^ however, partially ionized PAA does display a pH-dependent salting-out behavior,^38^ with UCST first reported by Ikegami et al.^39^ In addition, with multivalent salts, UCST behavior was observed with aqueous solutions of polyelectrolytes, such as PSS.^40,41^ Electrostatic interactions dominate the salting-out by multivalent counterions,^42^ as well as the pH-dependent UCST of NaPA described above.

In contrast, we speculate that the decrease in solubility of NaPA herein is due to solvent–polymer specific interactions,^43^ because the same trends are observed with the addition of either EG (ε = 39) or NMF (ε = 176), which leads to the opposite direction of changes in ε, *l*_B_ and electrostatic correlations as quantified by *l*_B_, and the Debye screening length (κ^–1^) for monovalent salt, κ^–1^ = (4π *l*_B_C_s_)^−1/2^. For example, at ϕ_EG_ = 0.25, ε = 72, and *l*_B_ = 0.79 nm, while at ϕ_NMF_ = 0.25, ε = 86, and *l*_B_ = 0.66 nm. We speculate that the addition of cosolvents decreases the degree of hydration around the free, pH-sensitive PA chains, rendering them more susceptible to the unfavorable interactions with the salty solvent and causing the PA to salt out.

To provide further physical evidence for the dual criticality, we elucidate which components are phase-separating using ^1^H NMR measurements on the separate upper supernatant and lower coacervate phases following an established protocol.^13^ ^1^H NMR allows for direct determination of the polycation and polyanion concentrations *separately*, which is not possible by cloud point or conventional thermogravimetric analysis. Figure 2 shows key features of the phase diagram of the NaPA/qPDMAEMACl complex with initial ϕ_EG_= 0.25, *c*_s_ = 0.45 mol/L and *c*_p_ = 0.10 mol/L along the temperature axis for the same sample as in Figure 1c. Three temperatures were selected above the LCST of 20 °C and two below the UCST of 7 °C to construct polymer concentration points on the binodal (Table S2 for the concentration data). As can be seen in the LCST branch (top), the supernatant and dense coacervate are composed of equimolar amounts of PA^–^ and qPDMAEMA^+^, within experimental uncertainty, as observed by the close proximity of each pair of black and red symbols. Furthermore, the associative nature is indicated by the fact that the polymer-rich phase (solid symbols) is rich in both PA^–^ and qPDMAEMA^+^, which are about 10 times the concentration of the initial starting polymer concentration.

**Figure 2:**
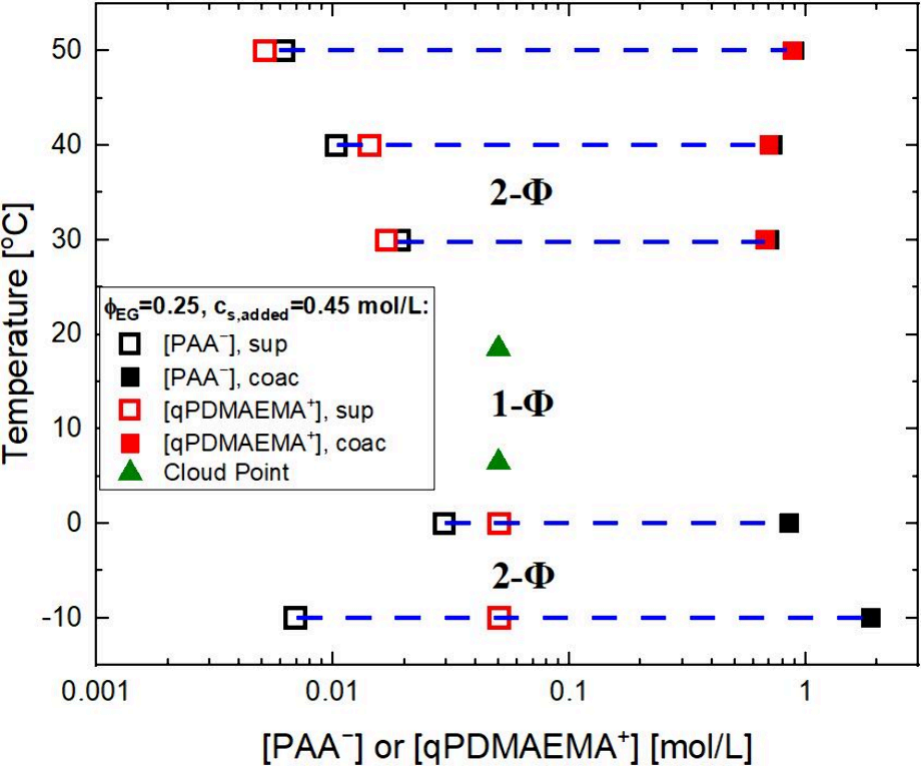
*T*–*c*_p_ phase diagram of NaPA/qPDMAEMACl complex with ϕ_EG_= 0.25, *c*_s,added_ = 0.45 mol/L and *c*_p_ = 0.10 mol/L, showing both LCST and UCST behaviors. The green triangles denote the two cloud point conditions for the same initial concentration of [PAA^–^]_0_ = [qPDMAEMA^+^]_0_ = 0.05 mol/L, with points on the binodal plane represented by the open and closed symbols as described in the legend for the polymer-poor and -rich phases.

Conversely, the UCST branch (bottom) shows a completely different scenario. The supernatant is rich in qPDMAEMA^+^ and poor in PA^–^, while the dense phase is only rich in PA^–^ and devoid of qPDMAEMA^+^ (therefore, there are no solid red symbols in the bottom branch). ^1^H NMR reveals the absence of qPDMAEMA^+^ in the dense phase via the absence of the characteristic methylene and methyl peaks corresponding to the −OCH_2_CH_2_N­(CH_3_)_3_^+^ structure (Figure S6). In the case of the UCST, the concentration of qPDMAEMA^+^ in the supernatant (open red symbols) is very close to the initial concentration of 0.05 mol/L in the PEC, because the dense phase only takes ∼2% of the total volume in these mixtures. A direct examination of Figure 2 based on the lever rule is provided in Figure S7, where the mass conservation was validated for each polyelectrolyte within an uncertainty of ±10%. Figure 3 shows the phase diagram of PEC with ϕ_NMF_ = 0.10, *c*_s_ = 0.49 mol/L and *c*_p_ = 0.10 mol/L, which also exhibits a similar behavior. In this case, the NMF/water mixture with ϕ_NMF_= 0.10 has a larger dielectric constant (ε = 82.2) than water (ε = 80) and than the EG/water mixture with ϕ_EG_ = 0.25 (ε = 72.1) at 23 °C, yet the segregative UCST behaviors exist only in the two cosolvent cases, not in pure water with intermediate ε. This points to a difference in the mechanisms underlying the UCST and LCST transitions, and the former is likely the consequence of less favorable polymer–solvent interactions in EG/water and NMF/water than in pure water.

**Figure 3:**
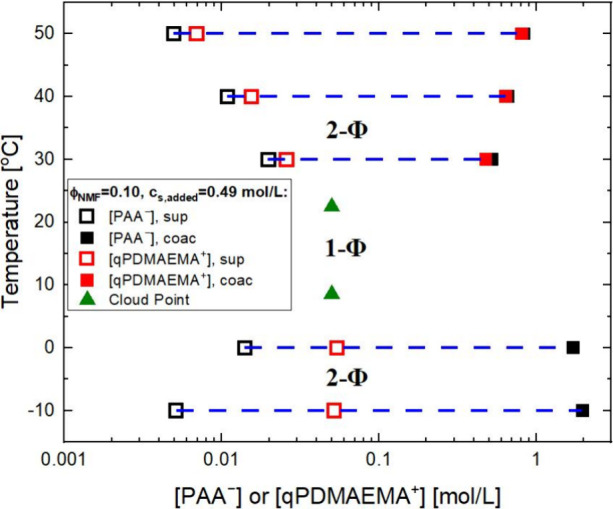
*T*–*c*_p_ phase diagram of NaPA/qPDMAEMACl complex with ϕ_NMF_= 0.10, *c*_s,added_ = 0.49 mol/L and *c*_p_ = 0.10 mol/L, showing both LCST and UCST behaviors. The green triangles denote the two cloud point conditions for the same initial concentration of [PAA^–^]_0_ = [qPDMAEMA^+^]_0_ = 0.05 mol/L, with points on the binodal represented by the open and closed symbols as described in the legend for the polymer-poor and -rich phases.

We investigated the effect of *c*_s_ to complement the cosolvent shift of the dual transition on a more traditional plot. In these experiments, for a given cosolvent concentration, we measured PECs with at least five *c*_p_ across two decades in molarity (0.01 to 1.0 mol/L). NaCl crystals were added stepwise to initial salt-free PECs. After each salt addition, the laser transmission % of the complex mixtures was monitored under stirring, where a criterion of transmission >50% was used to differentiate homogeneous 1-Φ region (open symbols, □) from phase-separated 2-Φ region (solid symbols, ■) solutions after equilibration. The results are shown in Figure 4. We refer to these as state diagrams to distinguish them from the binodal phase diagram of Figures 2 and 3. For clarity, Figure 4 shows only the phase boundaries that encompass the associative and segregative LLPS two-phase regions, as highlighted by red and purple shading, respectively. Notably in water, PEC shows only one phase boundary below 2 mol/L NaCl, consistent with our observation in Figure 1a and the previous publications by Ikegami et al.^39^ In fact, a small salting-out window appears at *c*_p_ ≈ 1.0 mol/L and *c*_s_ > 2.5 mol/L (Figure 4a), which is easily missed by previous studies due to the absence of measurements at such a high polyelectrolyte concentration. Such behavior was observed in weak polyelectrolyte and strong polyelectrolyte systems.^30,44^

**Figure 4:**
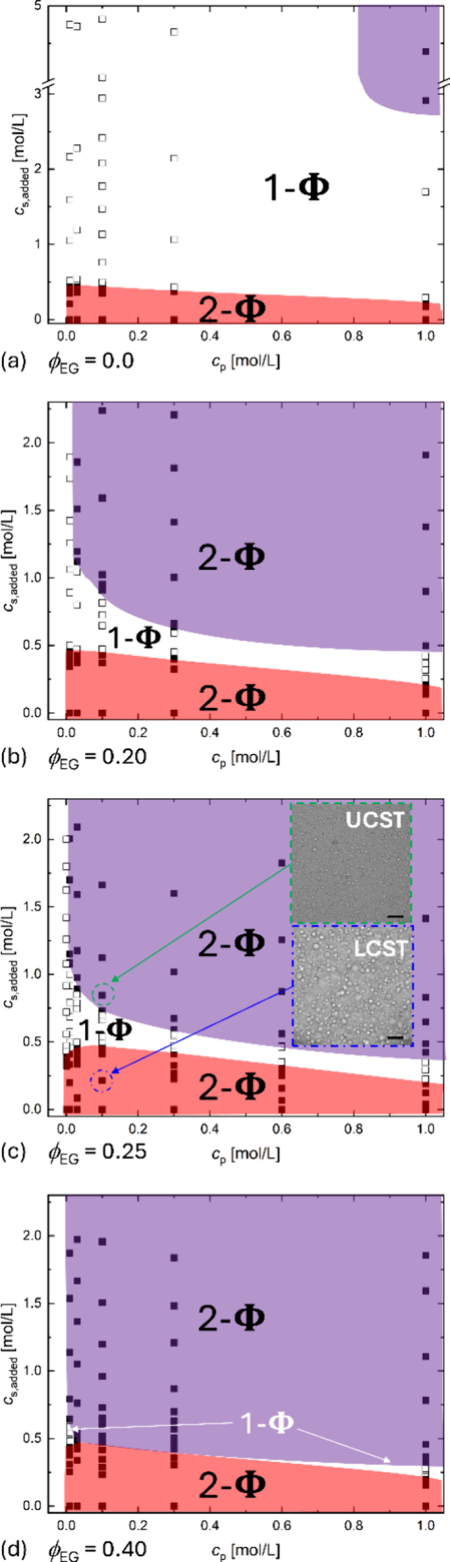
*c*_s_–*c*_p_ state diagrams of NaPA/qPDMAEMACl complexes with ϕ_EG_ ranging from (a) 0.0 to (b) 0.20, (c) 0.25, and (d) 0.40. The open and filled symbols denote the homogeneous and phase-separated solutions, respectively. In (c), optical microscopy images show droplets characteristic of LLPS below the upper critical and above the lower critical *c*_s_. Scale bar: 100 μm. In all cases, *T* is fixed at 20 °C.

Except for water, PECs in ϕ_EG_ ≥ 0.20 all show two phase boundaries within *c*_s_ ≤ 2 mol/L, with the lower 2-Φ region corresponding to associative LLPS and the upper 2-Φ region corresponding to segregative LLPS, as confirmed by ^1^H NMR. The *lower* boundaries appear insensitive to the cosolvent concentration, ϕ_EG_, which differs from observations with KPSS/PDADMAB complexes,^45^ where the critical salt concentration (salt resistance) was lowered by ∼25% at ϕ_EG_ = 0.25 and ∼39% at ϕ_EG_ = 0.50, while for NaPSS/poly­(vinylbenzyltrimethylammonium chloride) complexes^19^ the salt resistance was lowered by ∼60% at ϕ_EG_ = 0.20 (estimated from the optical microscopy images.)

The *upper* boundaries show a substantial lowering of the lower critical salt concentrations as ϕ_EG_ increases. At ϕ_EG_ = 0.20 (Figure 4b), both upper and lower critical salt conditions appear with boundaries that get closer at ϕ_EG_ = 0.25 (Figure 4c), which allows the dual transition to be traversed by changing temperature (Figure 1a and 1c). This is equivalent to going along the temperature axis perpendicular to the *c*_s_–*c*_p_ plane as shown in Scheme 1a, where increasing *T* can make an invariant reference point (•) go through both phase boundaries consecutively, essentially reproducing the transmission result in Figure 1b. Eventually at ϕ_EG_ = 0.40 (Figure 4d), the two miscibility gaps merge, revealing an hourglass-shaped diagram reminiscent of neutral polymer solutions^46,47^ and blends^48^ but with a very narrow 1-Φ region indicated by the arrow.

**Scheme 1:**
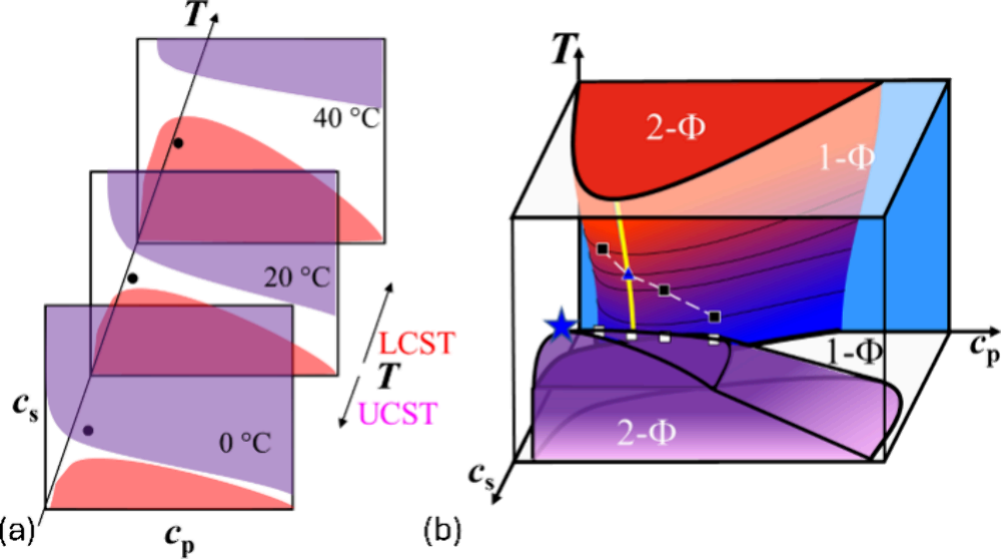
*T*–*c*_s_–*c*_p_ State Diagrams of NaPA/qPDMAEMACl in EG/Water (*ϕ*_EG_ = 0.25)^a^

a Panel (a) highlights a stack of three representative isothermal *c*_s_–*c*_p_ slices. With the increase of *T*, the associative two-phase region (red) expands while the segregative two-phase region (purple) shrinks. The solid symbols (•) are an invariant reference point guiding the eye. (b) Conceptual 3D *T*–*c*_s_–*c*_p_ state diagram of (a), comprised of two-phase envelopes. Notably, horizontally slicing the 3D cube from bottom to top recovers the stack of 2D *c*_s_–*c*_p_ diagrams in (a).

The temperature-dependent LLPS of symmetric mixtures of oppositely charged polyelectrolytes in a single solvent can be modeled beyond the VOT by including connectivity of charges and a single interaction parameter combined with a temperature-dependent solvent dielectric constant, where electrostatic-origin LCST behavior was recovered. However, the solvent quality (χ, Flory–Huggins interaction parameter) parametrization can give rise to multiple different behaviors. In particular, simultaneous UCST and LCST are possible, as shown by Adhikari et al. in Figure 2 of ref 32. In that work, applying a traditional inverse temperature dependence in χ and dipole–dipole interactions^32^ with strength proportional to *l*_B_^2^ can recover UCST and LCST. Linear strong and weak polyelectrolytes in aqueous solutions were studied for the effects of added multivalent salt that led to precipitation diagrams on the *c*_s_–*c*_p_ plane in some cases with reentrant behavior.^36^ The underlying phase separation often followed a UCST and could be modeled by a modified χ that considers short-ranged contributions from a screened Coulombic potential with strength proportional to κ^–2^.^49^

We considered if the cosolvent addition was reminiscent of the co-nonsolvency problem reported for hydrogen-bonding polymers such as poly­(*N*-isopropylacrylamide) (PNIPAAM) in aqueous solution. In that case, mixtures of two good solvents for the polymer lead to a nonmonotonic shift in the LCST. These well-defined studies found that mean-field ternary theory was insufficient to describe the LCST or chain collapse transition.^28,29,50^ Such effects are not immediately obvious with the present data, as cosolvent addition monotonically narrows the one-phase region and increases the LCST. Added salt further complicates the situation, as the solubility of salts can itself differ significantly between different solvents. Preferential cosolvent–polymer interactions in the form of preferential solvation are likely present, considering the appearance and substantial shift in the 2-Φ region for the segregative phase separation (purple-shaded area, Figure 4) of NaPA/qPDMAEMA, where NaPA experiences the poor solvent condition, but little if any shift in the 2-Φ region for the associative LLPS (red-shaded area, Figure 4), unlike the previously studied KPSS/PDADMAB complexes in which UCST was not observed.^45^ We do not have an adequate explanation for this system-dependent behavior but speculate that specific interactions between cosolvent and each polymer type, in particular PA, may lead to a compensation between driving forces for associated LLPS from electrostatic correlations and interaction parameters that minimizes the cosolvent effect. A homologous pair of oppositely charged polyelectrolytes could test this hypothesis.^8^

Finally, we examined the phase transition behaviors as shown by Figure 1a in a broader polymer concentration range, and the cloud points vs *c*_p_ at a fixed total salt concentration (i.e., *c*_s,added_ + *c*_s,counterion_) are shown in Figure 5. It is clear that increasing the ethylene glycol content in the cosolvents raises the LCST at all polymer concentrations. This is different than co-nonsolvency with PNIPAAM, where the cosolvent leads to a decrease in the LCST, followed by an abrupt increase.^28,50,51^

**Figure 5:**
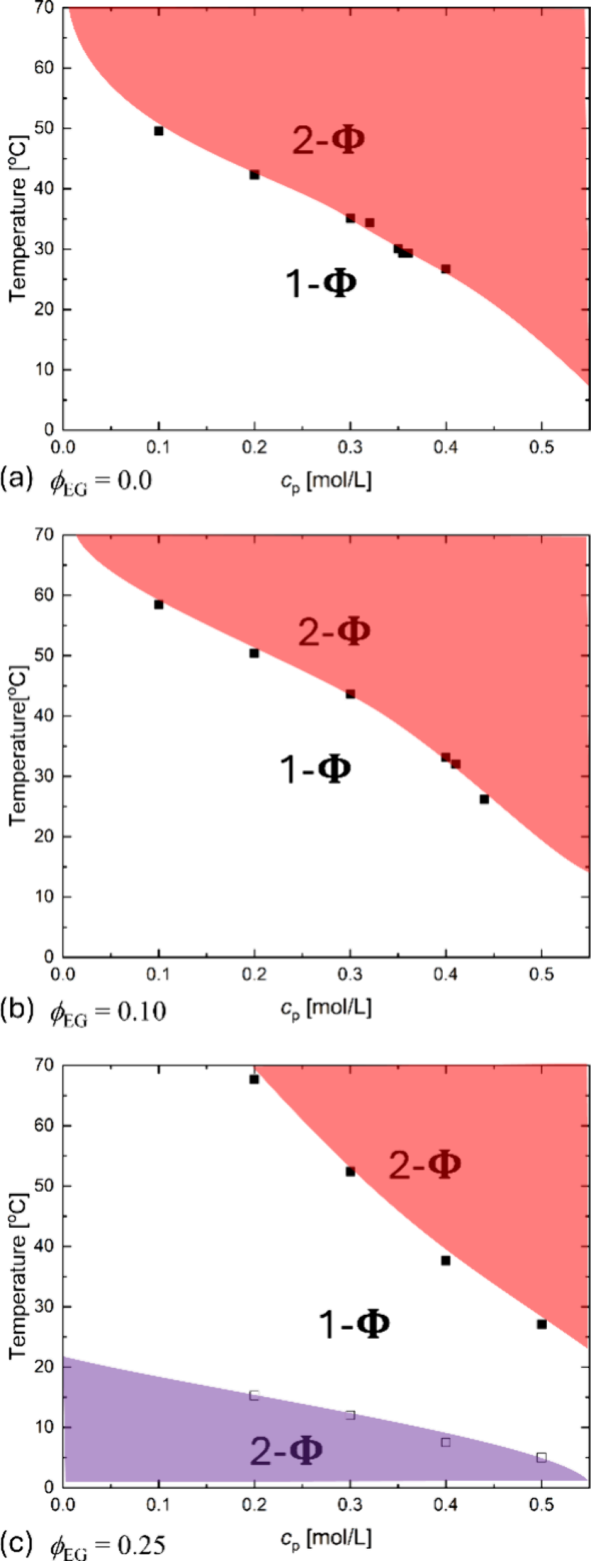
Cloud point on temperature–polymer concentration axis at fixed total salt concentration of 0.60 mol/L for (a) ϕ_EG_ = 0.0, (b) ϕ_EG_ = 0.10, and (c) ϕ_EG_ = 0.25.

In conclusion, the phase behaviors of a synthetic polyelectrolyte complex, NaPA/qPDMAEMACl, in aqueous cosolvent mixtures display a unique dual criticality with associative LCST LLPS (coacervation) and a segregative UCST LLPS (salting-out of NaPA) tuned by cosolvent fraction. The quantitative partitioning of NaPA polymer concentration was analyzed by ^1^H NMR, which supported the different phase separation outcomes. Such behavior was elucidated by the *c*_s_–*c*_p_ state diagrams, and the miscibility envelope was hypothesized to occur on a revised *T*–*c*_s_–*c*_p_ diagram^33^ (Scheme 1). In the case of a polycation and polyanion with different salting-out propensities, these results implicate that a single average polymer–solvent interaction parameter (χ) may not adequately reconcile the segregative LLPS that selectively partitions one polyelectrolyte at low temperature or high salt and preserves the physics of associative LLPS with stoichiometric complexes. Revisiting phase diagram calculations that include unique χ values for each polymer and solvent may be necessary to recover the dual criticality.

## Supplementary Material



## Notes

Materials and characterization by ^1^H NMR and X-ray photoelectron spectroscopy; methods for laser transmission, sample equilibration, phase extraction and concentration determination, and optical microscopy; lever rule results; tabulated binodal data; and additional UCST and LCST transition data for NMF cosolvent (PDF)
