# Supplementary material for: Cosolvent Control of Lower and Upper Critical Solution Behavior in Polyelectrolyte Complexes
Source: ACS Macro Lett. 2025 Jul 1;14(7):962–8. doi: 10.1021/acsmacrolett.5c00315 (PMC12269082; doi:10.1021/acsmacrolett.5c00315)
Supplement: Supplementary file 1 [file mz5c00315_si_001.pdf]

Supporting Information

Cosolvent control of lower and upper critical solution behavior in polyelectrolyte complexes

Yuanchi Ma[a,*] and Vivek M. Prabhu[b,*]

a. College of Polymer Science and Engineering, Qingdao University of Science and Technology, Qingdao, Shandong, China.
[b] Materials Science and Engineering Division, Material Measurement Laboratory, National Institute of Standards and Technology, 100 Bureau Drive, Gaithersburg, MD, United States.

Materials and Characterizations

Purification of poly(acrylic acid) (PAA):

Commercial PAA (Polymer Source, M_n = 15.5 kg/mol, M_w/M_n = 1.2) was diluted to 10 % solution and diafiltrated using an Amicon setup (Millipore-Sigma) equipped with a stirred cell, a selector valve and a reservoir to remove small-molecule acidic and ionic impurities. During the diafiltration, the filtrate passing through an ultrafiltration disc membrane (Ultracel, molecular weight cutoff = 3 kg/mol) was monitored until its ion conductivity decreases to the level of DI water (< 20 μS). The PAA solution retained in the stirred cell was then lyophilized to yield dry PAA solid, which was stored in a desiccator in a dark environment to avoid water adsorption and UV-crosslinking.

Synthesis and purification of quaternary poly(N,N-dimethylaminoethyl methacrylate chloride) (qPDMAEMACl):

qPDMAEMACl was synthesized by quaternization of the commercial PDMAEMA (Polymer Source, M_n = 35.0 kg/mol, M_w/M_n = 1.08) with methyl iodide (CH_3I, Sigma-Aldrich), followed by an ion exchange with excess NaCl (J.T.Baker). In a 500 mL round bottom flask, PDMAEMA (5.0 g, 31.8 mmol) was dissolved in 200 mL acetone, and CH_3I (14.2 g, 100 mmol) was added dropwise to the solution. The reaction was allowed to proceed for 48 h under vigorous stirring. The suspension after the reaction was centrifuged to remove the solvent and the majority of unreacted CH_3I, while the leftover solid (crude qPDMAEMAI) was washed with acetone for three times, and Soxhlet-extracted in reflux of acetone to remove organic impurities. The purified qPDMAEMAI was then dissolved in water to afford 10 % solution, washed with diethyl ether, and mixed with 1 L of 5 mol/L NaCl to achieve a $[Cl^-]/[I^-]$ ratio of at least 100/1. The resulting solution was concentrated, diafiltrated following the procedure described above, and lyophilized to give salt-free, dry qPDMAEMACl, of which the counterion purity was verified by X-ray photoelectron spectroscopy (XPS, Figure S2).

Other chemicals:

Ethylene glycol (EG, 99.5 %, analytical grade) was purchased from Thermo Fisher Scientific; N-methylformamide (NMF, 99 %) and sodium hydroxide (NaOH, ≥ 98 %, anhydrous pellets) were purchased from Sigma-Aldrich. These chemicals were used as received.

Sample preparation

To achieve this, purified PAA solid was first neutralized by a stoichiometric amount of freshly prepared sodium hydroxide solution with known molarity to afford concentrated NaPA solution, which was then diluted by milli-Q water to give 1.0 mol/L NaPA stock solution. The polycation was post-polymerization modified through a quaternization reaction of commercial PDMAEMA with methyl iodide, followed by an ion exchange with excess NaCl (detailed in SI). The dialyzed and dried qPDMAEMACl was also diluted to 1.0 mol/L as a stock solution. The PEC solutions with designated added salt concentration (c_s), polymer concentration (c_p) and cosolvent volume fraction (ϕ_{cosolv}) were then prepared and stored following a previously described protocol. The polymer characteristics are summarized in **Table S1**.

Table S1. Polymer Characteristics

	M_n [kg/mol]	N	M_w/M_n
NaPA[a]	15.5[c] (20.2[d])	215	1.2
qPDMAEMA-Cl[b]	35.0[c] (46.3[e])	223	1.08

[a]Synthesized by Reversible Addition-Fragmentation Chain Transfer (RAFT) polymerization (Polymer Source), then neutralized with NaOH. [b] Synthesized by anionic polymerization of *N*,*N*-dimethylaminoethyl methacrylate (Polymer Source), quaternized by CH_3I, counterion-exchanged and purified by ultrafiltration. [c]M_n – nominal number-average molar masses; N – degree of polymerization; and M_w/M_n – polydispersity are provided by the vendor. [d]Calculated by nominal M_n of the commercial PAA multiplied by the factor of (94/72), given 100% neutralization. [e]Calculated by nominal M_n of the commercial PDMAEMA multiplied by the factor of (207.5/157), supported by ^{1}H-NMR that shows quantitative quaternization (**Figure S1b**) and XPS that shows quantitative counterion-exchange (**Figure S2**).

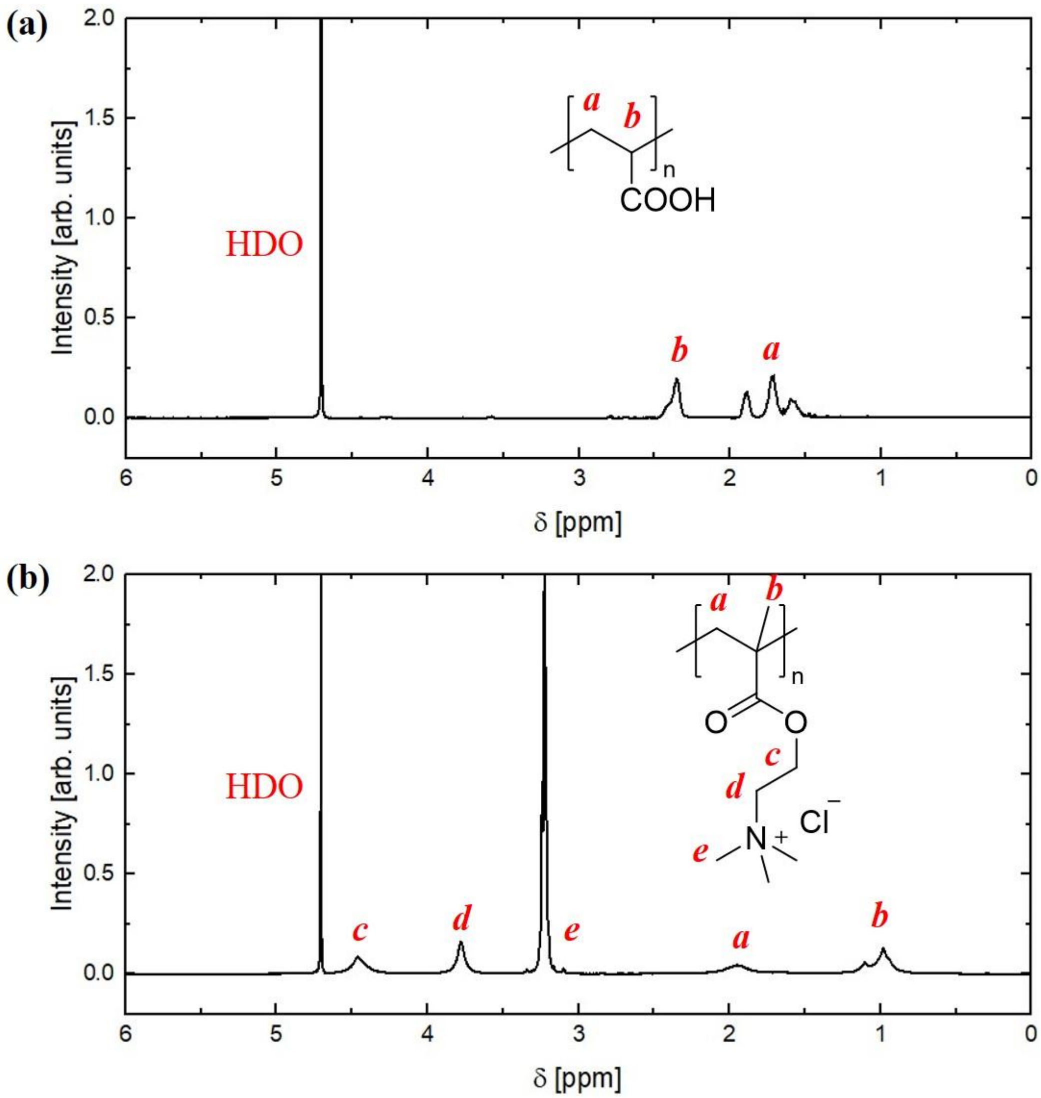

Figure S1. ^{1}H-NMR of the purified (a) PAA, and (b) qPDMAEMACl. All spectra were collected on a Bruker 600 MHz UltraShield spectrometer with deuterium oxide (D_2O) as the solvent unless otherwise noted. In (b), the single peak at ~ 3.2 ppm (denoted as *e*) indicates quantitative conversion from the $–N(CH_3)_2$ in PDMAEMA to the $–N(CH_3)_3^+$ in $qPDMAEMA^+$, whereas unreacted $–N(CH_3)_2$ would otherwise give rise to an additional peak between 2.0 and 2.5 ppm.[1]

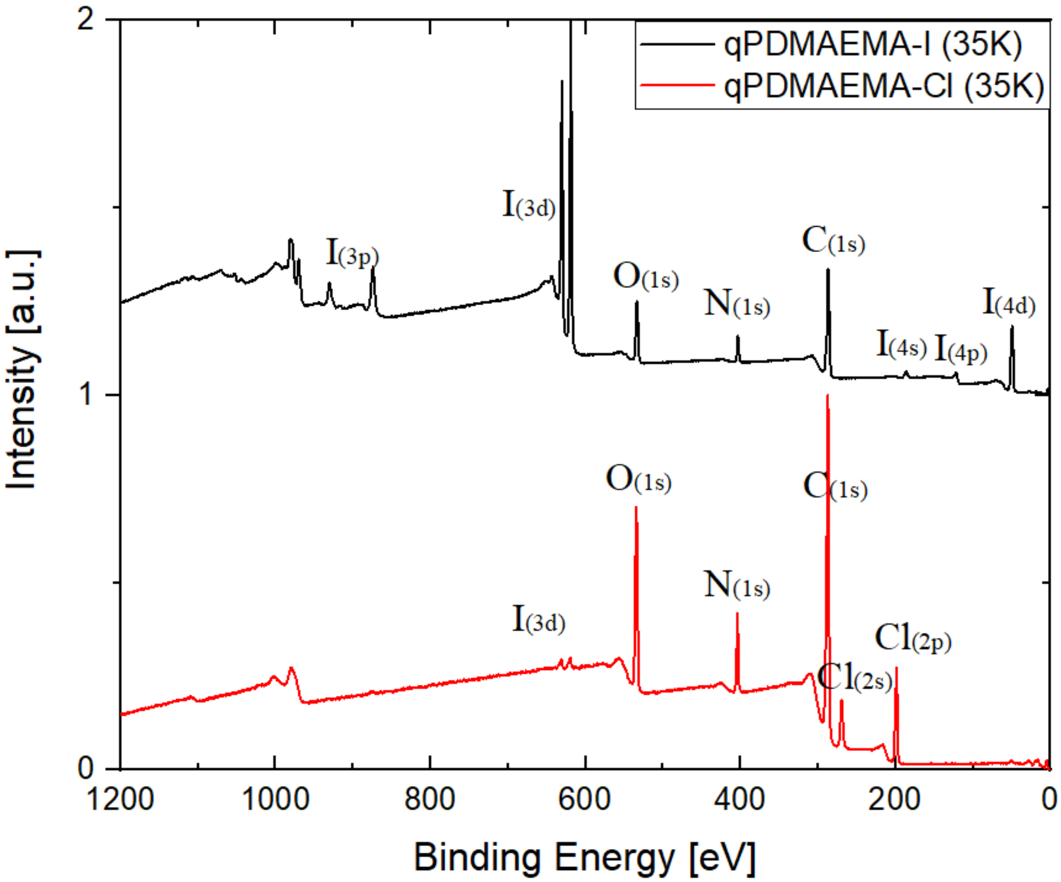

Figure S2. X-ray photoelectron spectroscopy (XPS) of qPDMAEMAI (black) and qPDMAEMACl (red), verifying complete exchange of counterions. All characteristic lines are labelled, with the subscripts denoting the corresponding electron configuration within atoms.

Experimental Methods

<u>Transmission measurements</u>

The transmission measurements of the polyelectrolyte complex (PEC) samples were conducted on a homemade setup with a 532 nm wavelength laser (Coherent VERDI), a set of neutral density filters, a sample heating stage (controlled by Quantum Northwest TC125 temperature controller, with precision of 0.01 °C), and a power meter (Thorlabs PM100D). During a temperature scan, the samples were heated at the rate of 1 °C/min under constant magnetic stirring, while the transmitted laser power as a function of time was recorded by the Thorlabs' power meter monitor software. The sample transmission was calculated by normalizing the transmitted laser power of the sample by that of an empty cuvette. The cloud point temperatures were defined by the inflection points of the transmission curves.

Equilibration and phase extraction

For the determination of binodal compositions, 1600 μL of PEC solution was used at each temperature. For lower critical solution temperature (LCST) samples at T = 30 ℃, 40 ℃ and 50 ℃, the Eppendorf tubes containing the PEC solution was equilibrated in a water bath with temperature control with precision of ±0.1 ° C (Isotemp, Fisher Scientific) for at least 2 h. For UCST samples with T = –10 ℃ and 0 ℃, the same setup was used despite replacing the water bath with water/ethanol to allow for sub-zero equilibration without freezing, which was much slower and took at least overnight, presumably due to the smaller density difference between the coexisting phases.

After the solution had phase-separated into two transparent phases with no visual droplets or cloudiness separated by a meniscus, the entire volume (~ 1500 μL) of the supernatant was extracted by pipette into a separate vial. Following the prompt removal of the residual supernatant, (20 – 40) μL of the coacervate was extracted using a gel tip to avoid perturbation to the interface.

Proton nuclear magnetic resonance (^{1}H-NMR)

A semiquantitative ^{1}H-NMR analysis[2] was used to determine [PA^-] and [$qPDMAEMA^+$] in our phase-separated samples, where the peaks corresponding to the two polyions were separately integrated and compared to that of the internal reference (R), sodium (3-trimethylsilyl)propionate -d_4 (TMSP-d_4). These [PA^-]/[R] and [$qPDMAEMA^+$]/[R] ratios were then corrected by those determined by a standard solution with known molarities of NaPA and qPDMAEMACl to obtain the actual polyelectrolyte concentrations in the unknowns.

^{1}H-NMR sample preparation follows the protocol described below. For a supernatant sample, 800 μL supernatant extract was vacuum dried and redissolved in 500 μL internal-referenced D_2O with [TMSP-d_4] = 5.088×10^{-3} M; the resulting solution was homogenous and can be directly used for measurements. In the case of coacervates, the dilution of vacuum-dried (20 – 40) μL coacervate extract into 500 μL internal-referenced D_2O resulted in partially phase-separated solutions, which were homogenized by adding ~ 15 mg NaCl crystals each, leading to negligible change in the overall sample volume.

The ^{1}H-NMR data were collected on a Bruker 600 MHz UltraShield spectrometer, and processed using the Bruker Topspin software version 3.6.1. For each Fourier-transformed spectrum, a baseline correction and a phase correction were performed consecutively before peak integration to ensure good reproducibility. As highlighted in Figure S3, for $qPDMAEMA^+$, the outstanding -$OCH_2C\underline{H}_2N(C\underline{H}_3)_3^+$ peaks at 3.9 ppm and 3.4 ppm were used for integration accounting for 11 protons altogether, while the methylene peak at 4.5 ppm was excluded due to its partial overlap with the solvent peak. The relative concentration of the polycation, [$qPDMAEMA^+$]/[R], is given by:

$$\frac{[\mathrm{qPDMAEMA}^+]}{[\mathrm{R}]} = \frac{S_{\mathrm{red}}/11}{S_{\mathrm{grey}}/9}.$$

For PA^- however, all its three ^{1}H-NMR-active protons reside in the same range as the five backbone protons of $qPDMAEMA^+$, therefore its relative concentration can only be calculated from the difference between the weighted blue- and red-shaded areas, following the equation:

$$\frac{[\mathrm{PA}^-]}{[R]} = \frac{(S_{\mathrm{blue}} - \frac{5}{11} S_{\mathrm{red}})/3}{S_{\mathrm{grey}}/9}$$

where S_{blue}, S_{red} and S_{grey} are the peak integration areas as illustrated in Figure S3.

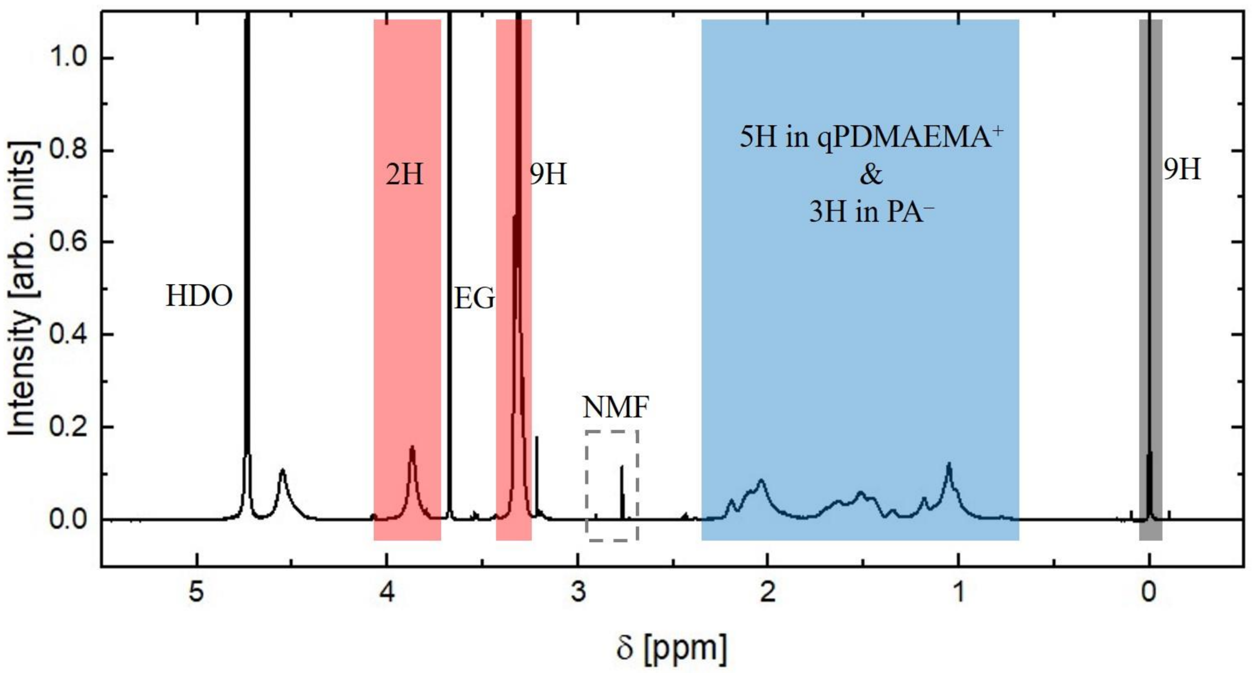

Figure S3. Representative ^{1}H-NMR spectrum of NaPA/qPDMAEMACl complex dissolved in internal-referenced D_2O. The peak integration areas were highlighted for the internal reference (in grey), $qPDMAEMA^+$ (in red), and for the overlapping region (in blue). The peaks of EG and NMF are the consequence of incomplete vacuum evaporation of the cosolvents, followed by vapor condensation.

<u>Optical Microscopy</u>

Bright-field optical-microscope images of coacervate droplets were obtained with an IX-71 Olympus microscope, with a 10x objective, and an Adimec 1000-M CCD camera. Droplets were initially freely suspended in solution as in the inset of Figure 4c in the main text and Figure S9, and eventually settled and adsorbed onto the glass slide. The field of view for these images is 742 micrometers.

Results

<u>Transmission of PECs in various solvents</u>

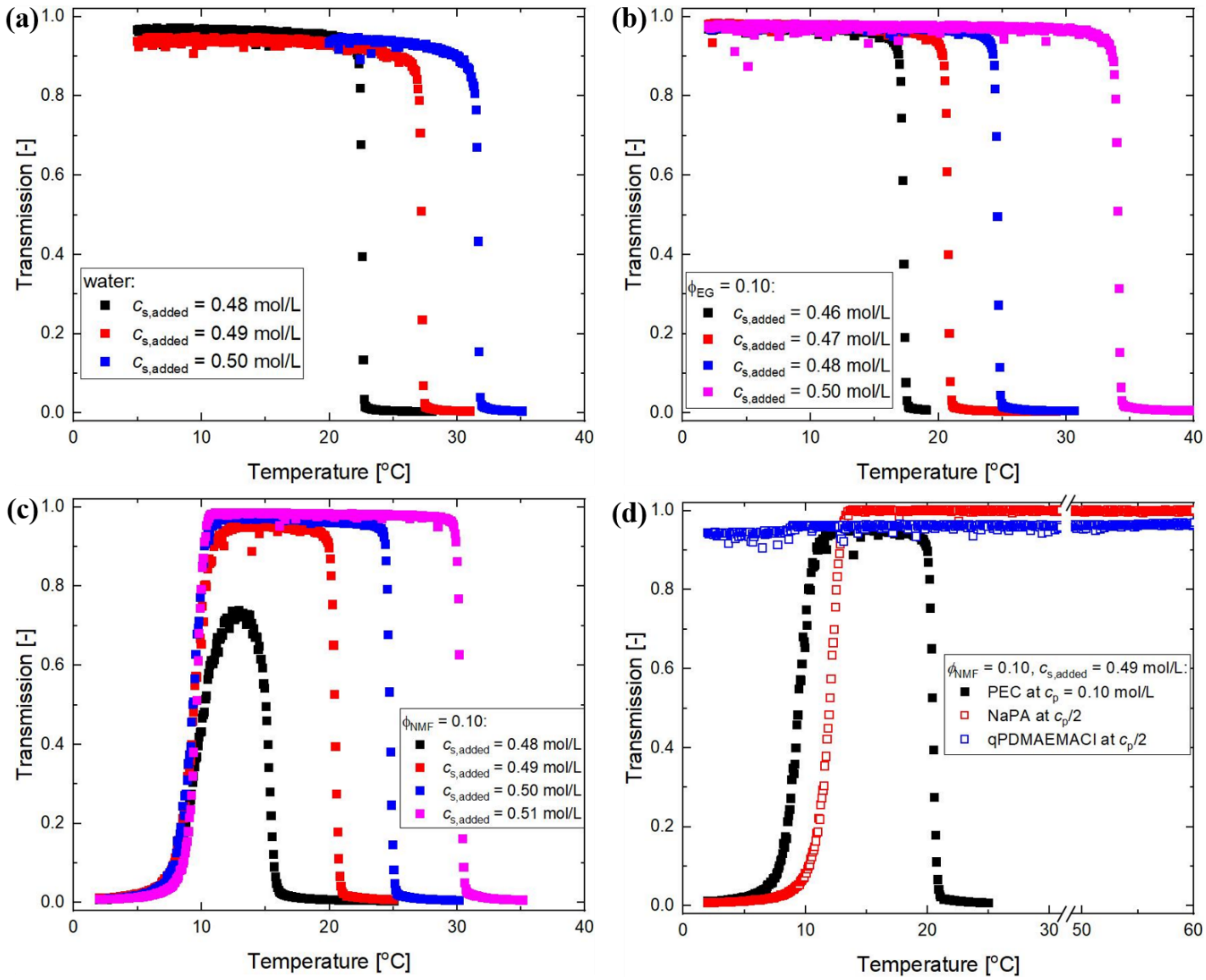

Figure S4. Transmission of NaPA/qPDMAEMACl complexes in (a) water; (b) ϕ_{EG}= 0.10; and (c) ϕ_{NMF}= 0.10. (d) The $c_{\mathrm{s,added}}$ = 0.49 mol/L PEC (red symbols) in (c) juxtaposed with NaPA and qPDMAEMACl at identical salt and solvent condition. In all cases, [PA$^-$] = [qPDMAEMA$^+$] = 0.05 mol/L.

Composition analysis of the binodal T-c_p phase diagrams

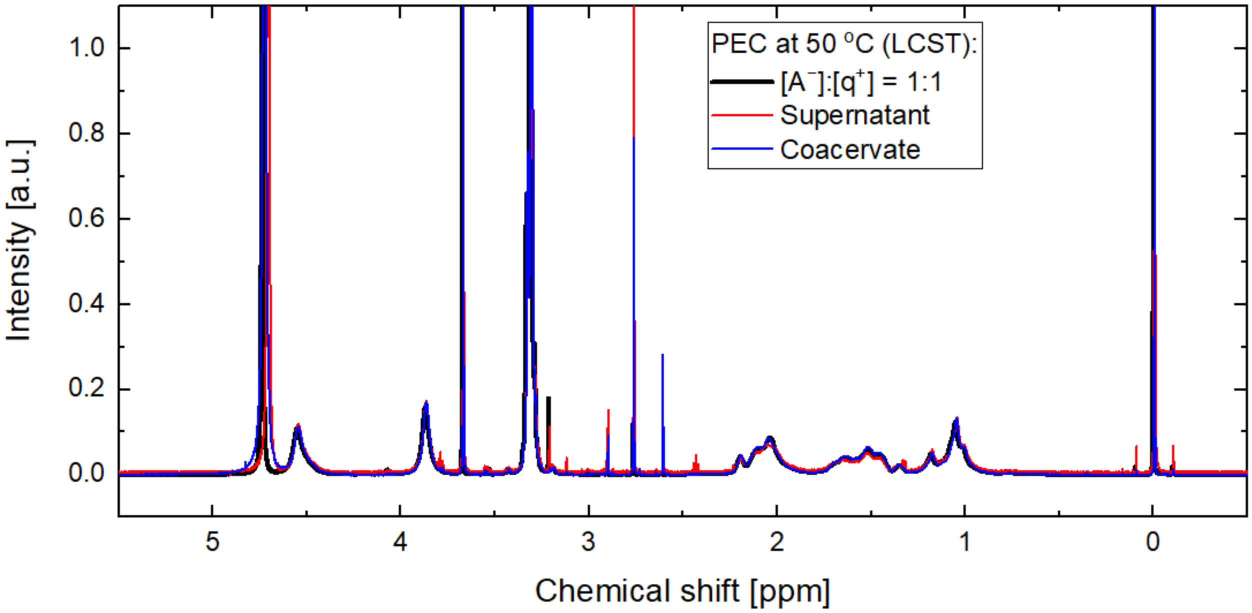

Figure S5. Overlaid ^{1}H-NMR spectra of the un-separated PEC (black), the LCST supernatant (red) and the LCST coacervate (blue). The supernatant and coacervate were extracted from the same sample that had been fully equilibrated at T = 50 °C. Intensities are arbitrarily renormalized to achieve maximum overlap at the two methylene peaks of $qPDMAEMA^+$ (4.5 ppm and 3.8 ppm), so as to compare and contrast the backbone peaks (2.2 ppm to 0.8 ppm). It is clear that in the LCST phase separation, $[PA^-]_{sup}/[qPDMAEMA^+]_{sup} \approx [PA^-]_{coac}/[qPDMAEMA^+]_{coac} \approx 1$.

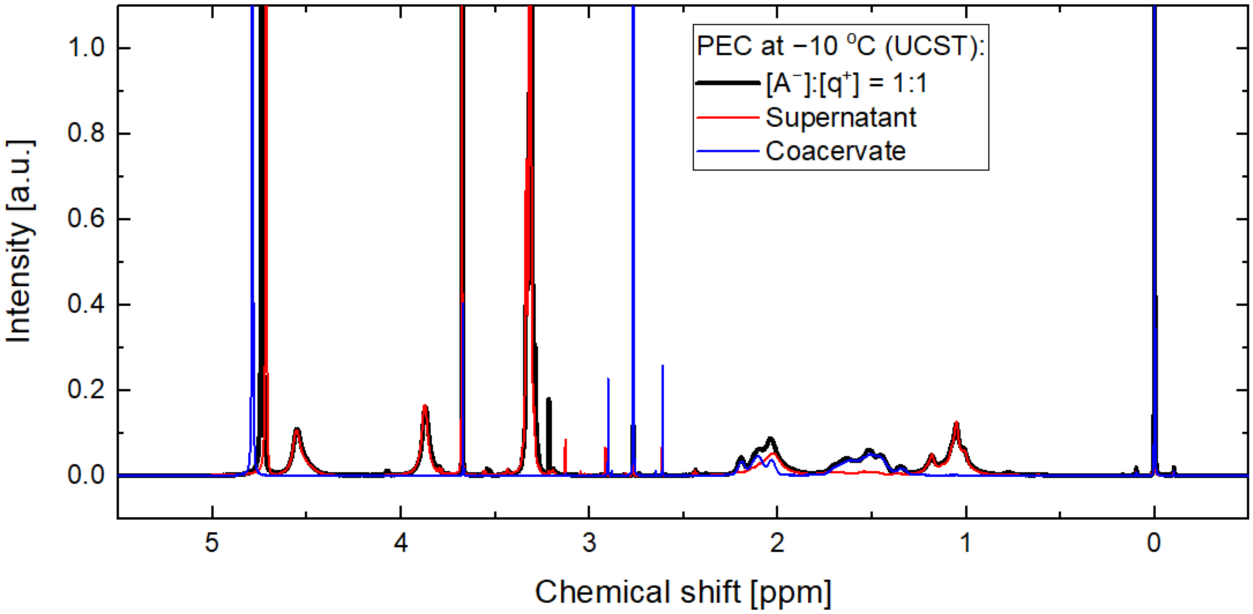

Figure S6. Overlaid ^{1}H-NMR spectra of the un-separated PEC (black), the UCST supernatant (red) and the UCST coacervate (blue). The supernatant and coacervate were extracted from the same sample that had been fully equilibrated at T = –10 ℃. Intensities are arbitrarily renormalized to achieve maximum overlap at the two methylene peaks of qPDMAEMA$^+$ (4.5 ppm and 3.8 ppm), so as to compare and contrast the backbone peaks (2.2 ppm to 0.8 ppm). Notably in the UCST phase separation, $[\text{qPDMAEMA}^+]_{coac}$ is below the detection limit of ^{1}H-NMR, while $[\text{PA}^-]_{sup}$ has a very small value, therefore giving the relation of $[\text{PA}^-]_{sup}/[\text{qPDMAEMA}^+]_{sup} \ll 1 \ll [\text{PA}^-]_{coac}/[\text{qPDMAEMA}^+]_{coac}$.

Table S2. Binodal composition and volume fraction as a function of *T* for the phase-separated PEC in ϕ_{EG} = 0.25.

T [°C]	ϕ_{coac} [a]	$[PA^-]_{sup}$ [mol/L]	$[qPDMAEMA^+]_{sup}$ [mol/L]	$[PA^-]_{coac}$ [mol/L]	$[qPDMAEMA^+]_{coac}$ [mol/L]
–10	0.0225	0.0069	0.0504	1.894	0
0	0.0225	0.0296	0.0505	0.851	0
30	0.0525	0.0190	0.0168	0.704	0.674
40	0.0525	0.0103	0.0143	0.731	0.700
50	0.0525	0.0062	0.0049	0.902	0.882

Note: (a) volume fraction of the coacervate phase (i.e., the dense phase) in a phase-separated PEC sample.

Examination of the mass conservation before and after phase separation serves as a sanity check of the data, which, based on the lever rule, should bear the following constraints:

$$(c_{x,sup}\,\phi_{sup} + c_{x,coac}\,\phi_{coac})/c_{x,0} = 1$$

where c are the concentrations of x = PA^- and $qPDMAEMA^+$, which are equivalent to $[PA^-]$ and $[qPDMAEMA^+]$ in the context albeit with a mathematical form. And obviously, we have $\phi_{sup} = 1 - \phi_{coac}$. The conservation of two polyions were examined separately, as shown in Figure S7.

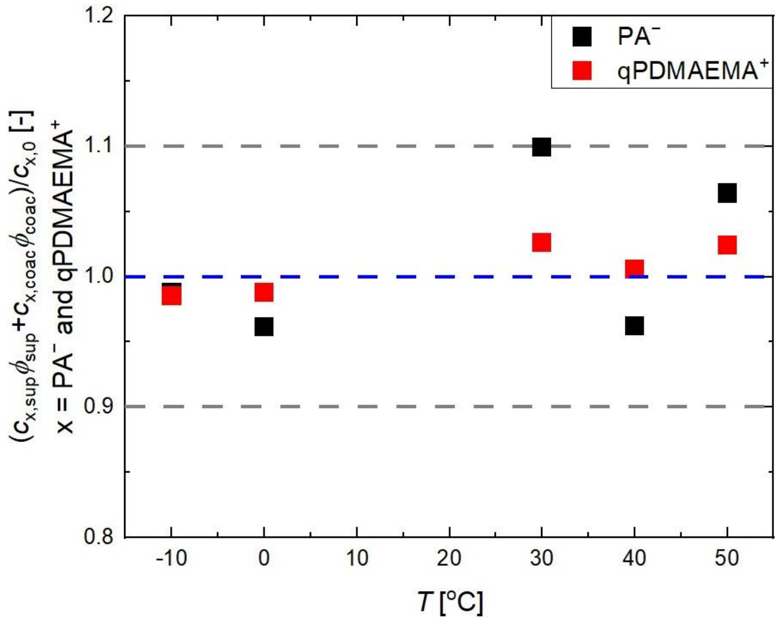

Figure S7. Examination of the mass conservation before and after phase separation at all *T*. The blue dashed line represents the theoretical value of unity, and the two grey dashed lines mark the relative error of ± 10 % to guide the eye.

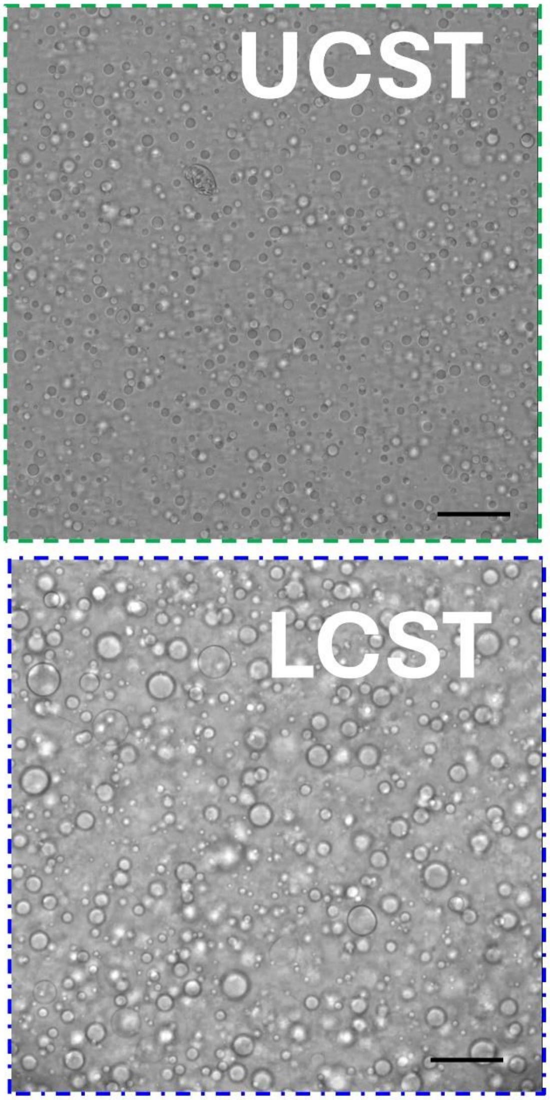

Figure S8. Enlarged **Figure 4c** optical microscopy images showing droplets characteristic of LLPS below the upper critical and above the lower critical c_s. Scale bar is 100 μm.

References

(1) De Jesús-Téllez, M. A.; Sánchez-Cerrillo, D. M.; Quintana-Owen, P.; Schubert, U. S.; Contreras-López, D.; Guerrero-Sánchez, C. Kinetic Investigations of Quaternization Reactions of Poly[2-(dimethylamino)ethyl methacrylate] with Diverse Alkyl Halides. *Macromol. Chem. Phys.* **2020**, *221*, 1900543.

(2) Ma, Y.; Ali, S.; Prabhu, V. M. Enhanced Concentration Fluctuations in Model Polyelectrolyte Coacervate Mixtures along a Salt Isopleth Phase Diagram. *Macromolecules* **2021**, *54* (24), 11338–11350.
